# Cardiopulmonary Resuscitation Outcomes of Patients with COVID-19; a One-Year Survey 

**DOI:** 10.22037/aaem.v9i1.1381

**Published:** 2021-11-04

**Authors:** Afshin Goodarzi, Masoud Khodaveisi, Alireza Abdi, Rasoul Salimi, Khodayar Oshvandi

**Affiliations:** 1 Department of Nursing, Hamadan University of Medical Sciences, Hamadan, Iran.; 2 Chronic Diseases (Home Care) Research Center, Department of Community Health Nursing, Hamadan University of Medical Sciences, Hamadan, Iran.; 3 Department of Nursing, School of Nursing & Midwifery, Kermanshah University of Medical Sciences, Kermanshah, Iran.; 4 Department of Emergency Medicine, School of Medicine, Besat Hospital, Hamadan University of Medical Sciences, Hamadan, Iran. Email:; 5 Mother and Child Care Research Center, Nursing and Midwifery School, Hamadan University of Medical Sciences, Hamadan, Iran.

**Keywords:** Cardiopulmonary resuscitation, Heart Arrest, COVID-19, Epinephrine

## Abstract

**Introduction:**

Assessing cardiopulmonary resuscitation (CPR) outcomes of patients with COVID-19 and employing effective strategies for their improvement are essential. This study is designed in this regard.

**Methods:**

This cross-sectional study was conducted between January 20, 2020 and January 20, 2021 in the emergency departments of two hospitals in Hamadan and Kermanshah, Iran. Participants were 487 patients with confirmed COVID-19 and cardiac arrest (CA) who had undergone CPR during the study period. Data were collected using the available CPR documentation forms developed based on the Utstein Style and analyses were performed using Chi-square, Fisher’s exact, and Mann-Whitney *U* tests and the logistic regression analysis.

**Results:**

Participants’ mean age was 69.31±14.73 years and most of them were male (61.8%) and suffered from at least one underlying disease (58.1%). The rate of total and in-hospital CA was 9.67% and 9.39%, respectively. The most prevalent first documented rhythm was asystole (67.9%) and the highest responsivity to CPR was for shockable rhythms. The rate of the return of spontaneous circulation (ROSC) was 9% and the rate of survival to hospital discharge was 2%. The significant predictors of CPR success were age (p = 0.035), epinephrine administration time interval (p = 0.00), CPR duration (p = 0.00), and First documented rhythm (p = 0.009).

**Conclusion:**

The rate of in-hospital CA among studied COVID-19 cases was 9.39% with 9% ROSC and 2% survival to hospital discharge rates after CPR. Primary CPR success among patients with COVID-19 was poor, particularly among those with asystole and bradycardia. It seems that old age and improper doses of epinephrine can reduce CPR success.

## 1. Introduction

Involvement of the cardiovascular system, particularly among patients with a history of cardiovascular disease, is one of the most serious complications of COVID-19 (1). Although COVID-19 is primarily manifested as a severe respiratory infection, different studies reported that it can cause stroke due to cerebrovascular ischemia, pulmonary artery thrombosis, spontaneous pneumothorax, cardiovascular disease, and type II diabetes mellitus due to the dysfunction of the pancreatic beta cells (2-6). Respiratory dysfunction and subsequent alteration of tissue oxygenation in patients with COVID-19 can directly affect the cardiovascular system and cause serious problems such as myocarditis, myocardial injuries, acute myocardial infarction, heart failure, cardiac dysrhythmia, and thromboembolism. These problems can lead to cardiac arrest (CA) (7). 

Studies on patients with COVID-19 show the increasing prevalence of in-hospital and out-of-hospital CA. For example, a study reported two times increase in the rate of out-of-hospital CA and reduced survival during the COVID-19 pandemic (8). A meta-analysis on four studies also indicated two times increase in the rate of in-hospital CA among patients with COVID-19 (9). Before the COVID-19 pandemic, in the U.S., an average of 292,000 cardiac arrests occurred annually (10, 11). There are no reliable statistics on CA rate in Iran; however, the average rates of CA in the United Arab Emirates and Saudi Arabia were respectively 11.7 and 7.76 cases per 1000 hospitalizations before the pandemic (12, 13). 

The American Heart Association noted that CPR for patients with COVID-19 is the same as CPR for patients without COVID-19 but recommended the use of personal protective equipment throughout CPR in order to reduce the risk of infection transmission (14). 

For instance, a study in China reported that the primary CPR success rate and thirty-day survival rate were 13.2% and 2.9%, respectively(15). Two other studies on out-of-hospital and in-hospital CA among patients with COVID-19 reported a survival to hospital discharge rate of zero percent (16, 17).

CPR outcomes depend on a wide range of factors such as cause of CA, underlying disease, the first documented rhythm, age, CA type (witnessed or unwitnessed), CPR duration, response time, call-to-arrival time, and adherence to CPR protocols (18-21). The lower CPR success rate among patients with COVID-19 has been attributed to factors such as the first documented rhythm (15, 16, 22). 

Despite the wide prevalence and the high mortality rate of COVID-19 throughout the world, there are limited reliable data about CPR and its outcomes among afflicted patients. This study aimed to assess the epidemiology and one-year outcomes of CPR among patients with COVID-19.

## 2. Methods

**2.1. Study design and setting**

This cross-sectional study was conducted on CPRs performed for patients with confirmed COVID-19 during the one-year period between January 20, 2020 and January 20, 2021 in emergency departments of Besat Hospital, Hamadan, Iran, and Imam Reza hospital complex, Kermanshah, Iran. The Institutional Review Board and the Ethics Committee of Hamadan University of Medical Sciences, Hamadan, Iran, approved the study protocol (codes: 9909186284 and IR.UMSHA.REC.1399.689). Necessary permissions for entering the study setting and performing data collection were obtained from the Research Administration of Hamadan and Kermanshah Universities of Medical Sciences, Hamadan and Kermanshah, Iran, and provided to the authorities of the study setting. Patients’ data were managed confidentially. It is noteworthy that in the study setting, consents for using patient data for research purposes were routinely obtained from patients and their family members at the time of hospital admission and were available via in-patient medical records.

**2.2. Participants**

Study population consisted of all patients with confirmed COVID-19, who had been hospitalized in the two mentioned hospitals and had undergone out-of-hospital or in-hospital CPR. Inclusion criteria were age over eighteen years, definite diagnosis of COVID-19 (based on PCR or PCR and high resolution computed tomography (HRCT), depending on the hemodynamic status), and out-of-hospital or in-hospital CA based on the Utstein Style criteria (23, 24). Patients with CA and no indication of CPR (i.e., those with rigor mortis or livor mortis) were not included. Patients with out-of-hospital CA and return of spontaneous circulation (ROSC) before hospital arrival who experienced another CA in emergency department were considered as out-of-hospital CA.

**2.3. Data collection**

The data collection instrument was the standard national CPR forms, which had been developed based on the Utstein Style and were routinely used for CPR documentation by CPR nurses in the study setting. The items on these forms include demographic characteristics, underlying disease, initial and final diagnoses, consciousness on arrival, CA type (in-hospital or out-of-hospital), out-of or in-hospital CPR, the first documented cardiac rhythms, use of defibrillation, necessary time for intravenous (IV) cannulation, administered medications during CPR, CPR duration, and CPR success. Data were collected from patients’ medical records. Hospital discharge status (dead or alive) was also assessed using the electronic medical record system of the study setting.

Based on the Utstein Style, the core CPR success outcomes are ROSC, post-CPR survival up to hospital discharge or for thirty days, and optimum neurological function up to hospital discharge or for thirty days. Complementary outcome based on this style is one-year survival after successful CPR (23). In this study, ROSC was considered as the primary outcome of CPR and post-CPR survival to hospital discharge was considered as the final outcome of CPR.

We defined adrenaline average dosing interval as the time between the first adrenaline dose and the resuscitation endpoint, divided by the total number of adrenaline doses received after the first dose. 

**2.4. Data analysis**

Data were analyzed using SPSS software (v. 20.0). The normality of age and CPR duration variables was tested using Kolmogorov-Smirnov test. Chi-square, Fisher’s exact, and Mann-Whitney *U* tests were used to assess the relationship of CPR outcomes with demographic characteristics, CPR time, CPR duration, epinephrine administration intervals, and IV cannulation time. Moreover, logistic regression analysis was performed to predict CPR outcomes. Variables with a significant relationship with CPR outcomes in univariable analysis were entered into the logistic regression model.

## 3. Results

***Baseline characteristics of studied cases***

During the one-year assessment period of the study, 5034 patients with COVID-19 had been hospitalized in the studied hospitals and 487 of them had experienced out-of-hospital or in-hospital CA. The mean age of patients with CA was 69.31±14.73 years and most of them were male (61.8%) and suffered from at least one underlying disease (58.1%). Baseline characteristics and CPR outcomes of the studied cases are presented in table 1. 

The total rate of CA among patients with COVID-19 was 9.67% and the total rate of in-hospital CA was 9.39%. Among patients with out-of-hospital CA, only 12.5% had been taken to hospital by the emergency medical services and had received CPR before hospital arrival. The most prevalent cardiac dysrhythmia was asystole (67.9%) and the mean CPR duration was 41.98 ± 8.98 minutes. The time interval between each two epinephrine administrations was 9.02 ± 4.31 minutes and in most cases (95.3 %) IV cannulation had been performed in less than one minute. The mean age of patients who experienced ROSC was 64.82±14.00 years, which was significantly less than the mean age in patients who had not experienced ROSC (69.76±14.74 years; Z=-2.464; p = 0.014). The mean age of patients who experienced survival to discharge was 64.50±9.11 years, and less than the mean age in patients who did not survive until discharge (64.91±15.26; Z=-0.533; p = 0.594). Also, the mean CPR duration of patients who experienced ROSC was 24.09±12.58 minutes, which was significantly shorter than the mean CPR duration in patients who had not experienced ROSC (43.77±6.17 minutes; Z=-9.716; p = 0.00), and the mean CPR duration of patients who experienced survival to discharge was 18.80±5.83 minutes, this time was 25.65±13.64 minutes in patients who did not survive to discharge (Z=-1.101; p = 0.271). The total rates of ROSC and survival to hospital discharge were 9.03% and 2.05%, respectively (Table 1). 

***Outcomes***

The ROSC outcome significantly correlated with participants’ age (p = 0.014), the first documented rhythm (p = 0.023), epinephrine administration time interval (p= 0.001), and CPR duration (p = 0.001) but survival to hospital discharge only had a significant relationship with the first documented rhythm (p = 0.042; Tables 2 and 3, respectively).

The results of the regression analysis showed age (p = 0.035), epinephrine administration time interval (p = 0.001), the first documented rhythm (p = 0.009), and CPR duration (p = 0.001) as the significant predictors of ROSC (Table 4).

**Table 1:** Baseline characteristics and outcomes of cardiopulmonary resuscitation in studied cases

**Variable**		**N (%)**	**ROSC**	Survival*
Gender	Male	301 (61.8)	31(10.30)	7 (2.32)
	Female	186 (38.2)	13 (6.99)	3 (1.61)
Type of CA	In-hospital	471 (96.7)	43 (9.13)	10 (2.12)
	Out-of-hospital	16 (3.3)	1 (6.25)	0 (0)
On-arrival status	Alert	242 (51.2)	25(10.33)	6 (2.48)
	Verbal	131 (27.6)	16(12.21)	4 (3.05)
	Painful	51 (10.8)	2 (3.92)	0 (0)
	Unresponsive	50 (10.5)	1 (2.0)	0 (0)
CPR time	08:00–14:00	131 (26.9)	15(11.45)	2 (1.53)
	14:01–20:00	126 (25.9)	12 (9.52)	5 (3.97)
	20:01–24:00	80 (16.4)	6 (7.5)	0 (0)
	00:01–07:59	150 (30.8)	11 (7.33)	3 (2)
Underlying disease	Hypertension	95(26.4)	11(11.58)	2(2.10)
	Diabetes mellitus	95(26.4)	10(10.53)	1(1.05)
	Cancer	34(9.4)	3(8.82)	0(0)
	IHD	70(19.4)	7(10)	3(4.29)
	CKD	15(4.2)	1(6.66)	1(6.66)
	COPD	10(2.8)	0(0)	0(0)
	Transplantation	2(0.5)	0(0)	0(0)
	CVA	6(1.7)	0(0)	0(0)
	Other	33(9.2)	1(3.03)	0(0)
First documented rhythm	Ventricular tachycardia	3 (0.6)	2 (66.66)	1 (33.33)
	Ventricular fibrillation	3 (0.6)	1 (33.33)	0 (0)
	Asystole	330 (67.9)	31 (9.39)	4 (1.21)
	PEA	3 (0.6)	0 (0)	0 (0)
	Bradycardia	147 (30.2)	10 (7.30)	5 (3.40)
Epinephrine administration Intervals	< 3 minutes	2 (0.4)	0 (0)	0 (0)
	3–5 minutes	66 (13.7)	23(34.85)	6 (9.09)
	> 5 minutes	414 (85.9)	21 (5.07)	4 (0.97)
Intravenous cannulation time	< 1 minutes	464 (95.3)	43 (9.27)	10 (2.15)
	>1 minute	23 (4.7)	1 (4.35)	0 (0)
Epinephrine delay	Yes	38(7.9)	2(5.26)	0(0)
	No	446(92.1)	42(9.42)	10(2.24)
Atropine	Yes	128(87.7)	9(7.03)	4(3.12)
	No	19(12.93)	1(5.26)	1(5.26)
Amiodarone	Yes	5(83.33)	3(60)	1(20)
	No	1(16.67)	0(0)	0(0)
Defibrillation	Yes	6(100)	3(50)	1(33.33)
	No	0(0)	(0)	0(0)
Airway management	Intubation	479(98.56)	43(8.98)	10(2.09)
	Mask	7(1.44)	1(14.28)	0(0)

Data are presented as number (%). CA: cardiac arrest; CPR: cardiopulmonary resuscitation; COPD: chronic obstructive pulmonary disease; CVA: Cerebrovascular accident; CKD: chronic kidney disease; IHD: Ischemic heart disease; PEA: pulseless electrical activity. * Survival to discharge.

**Table 2:** The relationships of participants’ characteristics with return of spontaneous circulation (ROSC)

**Variable**		**ROSC**	**p-value**
Gender	Male	31 (10.30)	0.216
	Female	13 (6.99)	
Type of CA	In-hospital	43 (9.13)	0.693
	Out-of-hospital	1 (6.25)	
On-arrival status	Alert	25 (10.33)	0.088
	Verbal	16 (12.21)	
	Painful	2 (3.92)	
	Unresponsive	1 (2.0)	
CPR time	08:00–14:00	15 (11.45)	0.632
	14:01–20:00	12 (9.52)	
	20:01–24:00	6 (7.5)	
	00:01–07:59	11 (7.33)	
Underlying disease	Yes	21 (7.72)	0.142
	No	23 (11.73)	
First documented rhythm	Ventricular tachycardia	2 (66.66)	0.023^*^
	Ventricular fibrillation	1 (33.33)	
	Asystole	31 (9.39)	
	PEA	0 (0)	
	Bradycardia	10 (7.30)	
Epinephrine administration Intervals	< 3 minutes	0 (0)	< 0.001^*^
	3–5 minutes	23 (34.85)	
	> 5 minutes	21 (5.07)	
Intravenous cannulation time	< 1 minutes	43 (9.27)	0.422
	>1 minutes	1 (4.35)	
Epinephrine delay	Yes	2(5.26)	0.393
	No	42(9.42)	
Atropine	Yes	9(7.03)	0.775
	No	1(5.26)	
Amiodarone	Yes	3(60)	1.00
	No	0(0)	
Defibrillation	Yes	3(50)	N/A^‡^
	No	(0)	
Airway management	Intubation	43(8.98)	0.488
	Mask	1(14.28)	

Data are presented as number (%).CA: cardiac arrest; CPR: cardiopulmonary resuscitation; PEA: pulseless electrical activity.^ ‡ ^not available. * Significant at level 0.05.

**Table 3:** The relationships of participants’ characteristics with survival to discharge

**Variable**		**Survival** ^†^	**p-value**
Gender	Male	7 (2.32)	0.971
	Female	3 (1.61)	
Type of CA	In-hospital	10 (2.12)	1.00
	Out-of-hospital	0 (0)	
On-arrival status	Alert	6 (2.48)	1.00
	Verbal	4 (3.05)	
	Painful	0 (0)	
	Unresponsive	0 (0)	
CPR time	08:00–14:00	2 (1.53)	0.194
	14:01–20:00	5 (3.97)	
	20:01–24:00	0 (0)	
	00:01–07:59	3 (2)	
Underlying disease	Yes	5 (1.84)	0.870
	No	5 (2.55)	
First documented rhythm	Ventricular tachycardia	1 (33.33)	0.042^*^
	Ventricular fibrillation	0 (0)	
	Asystole	4 (1.21)	
	PEA	0 (0)	
	Bradycardia	5 (3.40)	
Epinephrine administration Intervals	< 3 minutes	0 (0)	0.578
	3–5 minutes	6 (9.09)	
	> 5 minutes	4 (0.97)	
Intravenous cannulation time	< 1 minutes	10 (2.15)	1.00
	>1 minutes	0 (0)	
Epinephrine delay	yes	0 (0)	1.00
	no	10(2.24)	
Atropine	yes	4(3.12)	1.00
	no	1(5.26)	
Amiodarone	yes	1(20)	N/A^‡^
	no	0(0)	
Defibrillation	yes	1(33.33)	N/A^‡^
	no	0(0)	
Air way management	Intubation	10(2.09)	1.00
	Mask	0(0)	

Data are presented as number (%). CA: cardiac arrest; CPR: cardiopulmonary resuscitation. ^†^ Survival to discharge. ^‡ ^not available. * Significant at level 0.05.

**Table 4:** The predictors of cardiopulmonary resuscitation (CPR) outcomes

**Dependent**	**Independent **	**B**	**SE**	**Wald**	**df**	**p-value**	**OR**	**95** **%** ** CI**	
								**Lower **	**Upper **
ROSC	First documented rhythm (VT)	3.311	1.268	6.819	1	0.009^*^	27.40^†^	2.284	328.774
	Epinephrine interval (q/3–5min)	2.304	.342	45.394	1	< 0.001^*^	10.010^‡^	5.122	19.564
	CPR duration	-0.198	0.022	82.010	1	< 0.001^*^	.820	.785	.856
	Age	-0.021	0.010	4.442	1	.035^*^	.979	.960	.999
Survival to discharge	First documented rhythm (Asystole)	-1.910	1.512	1.594	1	.207	.148^¶^	.008	2.871

^†^. Reference level: Bradycardia;^‡^. Reference level: Epinephrine administration Intervals> 5 minutes;^ ¶^. Reference level: VT;* Significant at level 0.05. SE: standard error; OR: odds ratio. ROSC: return of spontaneous circulation; CI: confidence interval; VT: Ventricular tachycardia.

## 4. Discussion

The rate of in-hospital CA among studied COVID-19 cases was 9.39% with 9% ROSC and 2% survival to hospital discharge rates after CPR. Primary CPR success among patients with COVID-19 was poor, particularly among those with asystole and bradycardia. It seems that old age and high or low doses of epinephrine can reduce CPR success.

In line with this finding, a previous study reported that the rate of in-hospital CA among patients with COVID-19 was 10% (9).

Most participants suffered from at least one underlying disease, particularly diabetes mellitus, hypertension, cardiovascular disease, and cancer. A meta-analysis also reported the prevalence of different underlying diseases among patients with COVID-19 (25). Affliction by underlying diseases increases mortality rate among patients with COVID-19 (25, 26). Compromised immunity due to diabetes mellitus, decreased inflammatory cytokines among patients with cardiovascular disease, or chemotherapy among patients with cancer is considered as a major risk factor for affliction by COVID-19 (27, 28). On the other hand, findings showed that 41.9% of participants had no underlying disease, denoting the high prevalence of COVID-19 among people with no underlying disease. These findings question the widespread belief that COVID-19 affects people with no underlying disease less frequently. The high transmissibility of the virus is a significant factor contributing to the high prevalence of COVID-19 even among people with no underlying disease.

Primary CPR success, i.e., ROSC, was observed among only 9% of the patients with COVID-19 who had experienced CA. CPR success rate among patients with in-hospital CPR was also higher than patients with out-of-hospital CPR. A previous study in this area reported that the rate of ROSC after CPR was 25.9% for out-of-hospital CA and 30.6% for in-hospital CA (16). Moreover, a meta-analysis on four studies on 621 patients with COVID-19 showed that the pooled prevalence of primary CPR success was 39% (95% CI: 21.0%–59.0%) (9). The rate of primary CPR success in the two mentioned studies are much better than the rate in our study. Comparison of the findings of the present study with the findings of two previous studies in Iran before the COVID-19 pandemic also reveals a lower CPR success rate among patients with COVID-19 (21, 29). This lower CPR success rate can be attributed to the higher prevalence of asystole in the present study compared with previous studies on patients with and without COVID-19 (16, 17, 29, 30). Asystole is less responsive to CPR than other shockable dysrhythmias. Another reason for the lower CPR success rate in the present study may be non-adherence to epinephrine administration protocols. Some studies also reported that poor CPR outcomes among patients with COVID-19 may be due to the employment of novice staff for CPR during the COVID-19 pandemic, delayed CPR onset due to the need for using personal protective equipment, and CPR staff’s concern over affliction by COVID-19 during CPR (31, 32). Delayed or slow CPR onset and subsequent CPR prolongation have significant negative relationships with CPR outcomes (33-35). The lower rate of CPR success among patients with out-of-hospital CA in the present study may also be due to the fact that only 12.5% of them had been taken to hospital by emergency medical services and the others had been transported using private vehicles or taxi and hence, had not received out-of-hospital CPR. A previous study had also reported the same finding (36).

Study findings showed that only 2% of patients had survival to hospital discharge. All these patients had experienced in-hospital CA. A meta-analysis on patients with COVID-19 and in-hospital CA also reported that the cumulative prevalence of survival to discharge rate was 3% (9), while in two other studies none of the patients with COVID-19 and in-hospital CA had survived to hospital discharge (16, 30). COVID-19 significantly affects different body organs and hence, CA among afflicted patients is mostly fatal. Therefore, preventive measures, timely treatments, and careful monitoring of critically-ill patients with COVID-19 are necessary to prevent the occurrence of CA.

Findings revealed that primary CPR success a had significant relationship with age, the first documented rhythm, epinephrine administration interval, and CPR duration, while final CPR success had a significant relationship only with the first documented rhythm. The logistic regression analysis revealed age, epinephrine administration interval, the first documented rhythm, and CPR duration as the significant predictors of primary CPR success. The mean CPR duration was 24 minutes (with an interquartile range of 15–30) for patients with successful primary CPR and 43 minutes for unsuccessful CPR. The mean CPR duration was six minutes (with an interquartile range of 4–14) among patients with successful primary CPR and in-hospital CA in a previous study (22) and eight minutes (with an interquartile range of 4–10) in another study (17). The longer CPR duration in the present study compared with previous studies may be due to the fact that the study included patients with out-of-hospital CA. A study reported that there is no maximum time for CPR efforts, while longer CPRs were associated with greater survival to discharge rate (37). Although the mean CPR duration among survived patients in the present study was shorter, 6.8% of successful CPRs had lasted more than 45 minutes, denoting that CPR prolongation can be a determining factor in CPR success. 

The mean of participants’ age in the present study was 69 years and the mean age among participants with successful CPR was significantly less than those with unsuccessful CPR. The results of a meta-analysis on more than half a million patients with COVID-19 from different countries also reported age as a significant predictor of mortality (38). These findings highlight the importance of timely preventive measures for older patients to improve treatment outcomes among them because they are less responsive to treatments in critical conditions such as CA. 

Epinephrine administration interval was one of the significant predictors of primary CPR success in the present study. This interval was 5.41±1.74 minutes for patients with successful primary CPR. The rates of primary CPR success and survival to hospital discharge were 34.85% and 9.09%, respectively, for patients who had received epinephrine every 3–5 minutes, and 5.07% and 0.97% for patients who had received it in intervals longer than five minutes. Moreover, none of the patients who had received extra high doses of epinephrine (i.e., with intervals less than three minutes) had experienced successful primary CPR and survived to hospital discharge. The standard dose of epinephrine for adults is 1 milligram every 3–5 minutes throughout CPR (39). Our findings showed that CPR success among patients who had received high doses of epinephrine was less than those who had received it in doses less than the recommended standard dose. Although poor CPR outcomes among patients with COVID-19 can be attributed to COVID-19 severity, the role of high doses of epinephrine in causing cytokine storms should be taken into account. Further studies are needed to assess this role and the necessity to use safer medications instead of epinephrine for the CPR of patients with COVID-19.

The most prevalent first documented cardiac rhythms among study participants were asystole and bradycardia, respectively, and the prevalence of shockable dysrhythmias was 1.24%. The prevalence of shockable dysrhythmias in five earlier studies on patients with COVID-19 was 3.7%–13%, which is less than the rate among patients without COVID-19 (15-17, 22, 30). Pulmonary involvement and its associated hypoxia may be a reason for the lower rate of shockable dysrhythmias among patients with COVID-19.

The highest rate of successful primary CPR and survival to discharge rate were among patients with pulseless ventricular tachycardia. The first documented rhythm was a significant predictor of primary CPR success in the present study. Studies on patients without COVID-19 (40, 41) and a meta-analysis on patients with COVID-19 found poorer CPR outcomes for patients with non-shockable dysrhythmias (9). Despite the lower prevalence of shockable dysrhythmias among patients with COVID-19, these dysrhythmias have better prognosis than non-shockable dysrhythmias. 

Although the Primary CPR success rate in our study was lower than the results reported by other studies in this area, the survival to hospital discharge rate in this study was at an acceptable level compared to other studies. The prevalence of shockable dysrhythmias among patients with COVID-19 is also much lower than patients without COVID-19, resulting in lower responsiveness to CPR among patients with COVID-19. Old age and high doses of epinephrine are factors that can negatively affect CPR outcomes, particularly primary CPR success, among patients with COVID-19. Further studies are needed to assess the effects of epinephrine administration on CPR outcomes among these patients. Although the mean CPR duration among survived patients in the present study was shorter, the increase in resuscitation time was associated with an increase in the number of survivors. On the other hand, by comparing the results of the present study with studies conducted in Iran before the epidemic, and in non-COVID patients, it can be claimed that the outcomes of resuscitation are weaker in patients with COVID-19. Poor CPR outcomes among patients with COVID-19 highlight the importance of exploring CPR staff’s experiences and the effects of their concerns over affliction by COVID-19 on CPR quality and outcomes.

## Limitation

Our study had limitations; in some cases, some information related to the resuscitation process was not available in resuscitation registration forms and patient records. It is possible for some information to be incorrectly recorded by CPR staff, and these limitations were beyond the control of the researchers.

## 5. Conclusion

The rate of in-hospital CA among studied COVID-19 cases was 9.39% with 9% ROSC and 2% survival to hospital discharge rates after CPR. Primary CPR success among patients with COVID-19 was poor, particularly among those with asystole and bradycardia. It seems that old age and high or low doses of epinephrine can reduce CPR success.

## 7. Declarations

### 7.1. Availability of data

The datasets used and/or analyzed during the current study are available from the corresponding author on reasonable request.

### 7.2. Competing interests

The authors declare that they have no competing interests

### 7.3. Funding

Financial resources for the design of the present study and collection, analysis, and interpretation of data and writing the manuscript are provided by Hamadan University of Medical Sciences.

### 7.4. Authors' contributions

Study design: AG, KO; Data gathering: AG, RS; Analysis: AG, ARA; Interpreting: AG, , MK, ARA; Drafting: AG, KO; Critically revised the paper: All authors.

### 7.5. Acknowledgement

We would like to thank the Research Administration of Hamadan University of Medical Sciences, Hamadan, Iran, as well as the authorities, nurses, and medical records staff of the study setting who helped us conduct this study.

## 6. List of abbreviations

CPR: Cardio Pulmonary Resuscitation, CA: Cardiopulmonary Arrest, IV: Intravenous, ROSC: Return of Spontaneous Circulation
